# EEG Frequency Bands in Psychiatric Disorders: A Review of Resting State Studies

**DOI:** 10.3389/fnhum.2018.00521

**Published:** 2019-01-09

**Authors:** Jennifer J. Newson, Tara C. Thiagarajan

**Affiliations:** Sapien Labs, Arlington, VA, United States

**Keywords:** EEG, electroencephalography, resting-state, power spectrum, psychiatric, ADHD, schizophrenia, depression

## Abstract

A significant proportion of the electroencephalography (EEG) literature focuses on differences in historically pre-defined frequency bands in the power spectrum that are typically referred to as alpha, beta, gamma, theta and delta waves. Here, we review 184 EEG studies that report differences in frequency bands in the resting state condition (eyes open and closed) across a spectrum of psychiatric disorders including depression, attention deficit-hyperactivity disorder (ADHD), autism, addiction, bipolar disorder, anxiety, panic disorder, post-traumatic stress disorder (PTSD), obsessive compulsive disorder (OCD) and schizophrenia to determine patterns across disorders. Aggregating across all reported results we demonstrate that characteristic patterns of power change within specific frequency bands are not necessarily unique to any one disorder but show substantial overlap across disorders as well as variability within disorders. In particular, we show that the most dominant pattern of change, across several disorder types including ADHD, schizophrenia and OCD, is power increases across lower frequencies (delta and theta) and decreases across higher frequencies (alpha, beta and gamma). However, a considerable number of disorders, such as PTSD, addiction and autism show no dominant trend for spectral change in any direction. We report consistency and validation scores across the disorders and conditions showing that the dominant result across all disorders is typically only 2.2 times as likely to occur in the literature as alternate results, and typically with less than 250 study participants when summed across all studies reporting this result. Furthermore, the magnitudes of the results were infrequently reported and were typically small at between 20% and 30% and correlated weakly with symptom severity scores. Finally, we discuss the many methodological challenges and limitations relating to such frequency band analysis across the literature. These results caution any interpretation of results from studies that consider only one disorder in isolation, and for the overall potential of this approach for delivering valuable insights in the field of mental health.

## Introduction

In 2001 the World Health Organization^1^ (WHO) reported that about 450 million people worldwide suffer from some form of mental disorder or brain condition, and that 1 in 4 people will meet this criteria at some point in their life (Sayers, 2001). More recent statistics^2^ suggest that globally, 300 million people are affected by depression, 60 million people suffer from bipolar disorder, 23 million people are affected by schizophrenia, 1 in 160 children has autism spectrum disorder^3^ and between 5% and 7% of children and adolescents suffer from attention deficit-hyperactivity disorder (ADHD; Polanczyk et al., 2007). Diagnosis of these psychiatric disorders is typically carried out using clinical interviews structured around the diagnosis classification systems of DSM-5 and ICD-11. These diagnostic criteria are based on self-reported symptom clusters, with each disorder type having its own group of symptoms which can include behavioral, cognitive, affective or physical disturbances. For example, ADHD diagnosis primarily focuses on cognitive and behavioral complaints by the child or adult, whilst diagnosis of depressive disorders typically focuses on disruptions to an individual’s affective and physical functioning.

However, the reliance on a subjective assessment approach which can be prone to patient and expert bias means that researchers have been trying to develop new ways to inform clinical diagnosis and treatment effectiveness using objective symptom biomarkers, with electroencephalography (EEG) being one method of interest (McLoughlin et al., 2014; Jeste et al., 2015; Olbrich et al., 2015). The approach that dominates the literature focuses on analyzing broad frequency bands in the EEG power spectrum termed delta, theta, alpha, beta, and gamma (Berger, 1929; Jasper and Andrews, 1936; Hoagland et al., 1937a,b; Dustman et al., 1962). This interpretation of the EEG signal in terms of spectral bands has its origins in the technical limitations of the pre-computer era of the 1930s and ‘40s when few other analytical options were available. However, this approach results in a reduction in the rich temporal information available within the EEG and was, even at that time, acknowledged to be sub-optimal (Walter, 1938). Yet, despite the tremendous progress in computing power and available algorithms, the spectral band approach continues to persist as the dominant approach to EEG analysis, including in the development of clinical biomarkers. A recent example of this is the approval by the FDA^4^ of the use of the theta/beta ratio as a biomarker for ADHD diagnosis (Saad et al., 2015; Gloss et al., 2016) whilst others are exploring the application of alpha-asymmetry as a potential marker for depression (van der Vinne et al., 2017; Kaiser et al., 2018). One question, therefore, is whether the approach of splicing the power spectrum into bands has persisted because it offers a superior approach in terms of research insight, methodological standardization, and reliability of results across studies, or whether it is because researchers have simply kept with the status quo of 80 years ago.

To explore the degree to which spectral band analysis of the EEG offers a reliable and useful approach for understanding different psychiatric disorders, we have reviewed the methods and results from 184 resting-state EEG studies across a host of psychiatric disorders that report differences (or lack thereof) in the various frequency bands within the power spectrum. The objectives of this review are therefore threefold. First, to determine the dominant patterns of results and reveal similarities and dissimilarities in the spectral trends both between and within different brain disorders during resting-state; second, to report the reliability and consistency of results across disorder types to determine the validity of applying power spectral analyses to inform on individual psychiatric disorders; and thirdly to review the methodological and analytical approaches across all studies to determine the degree to which they can be compared and contrasted to draw reliable conclusions within the field. In this respect, we provide an objective view of the literature along numerous methodological dimensions from sample size and choice of demographic (e.g., age, gender) to method of clinical diagnosis and parameters of EEG recording (e.g., reference type) and analysis (e.g., artifact removal, Fourier transform algorithm) used both within and across disorder types. We note that we restrict our focus to analysis of frequency bands at the level of single channels or averaged across channels and do not cover derivative analysis of these spectral bands such as their spatial coherence or asymmetry.

Such a cross disorder view is particularly warranted since the majority of clinical resting-state EEG studies focus primarily on one clinical disorder at a time, and do not offer a perspective across a broader range of psychiatric disorders. Therefore, whilst a study may report changes in particular frequency bands for one disorder type, it is not always obvious whether this is unique to this particular disorder, or whether similar patterns of change are found across other psychiatric disorders. In other words, are there unique EEG signatures which differentiate one disorder from another, or do the macro-level changes observed in studies employing a frequency band approach overlap with other disorders, therefore being more limited in their clinical diagnosis potential.

## Materials and Methods

### Studies Identified and Reporting Characteristics

We present a review of studies published over the last 25 years that report spectral power in different bands during resting state conditions (eyes open and/or closed) across 10 mental health disorders. These include depression, bipolar disorder, addiction, autism, ADHD, anxiety, panic disorder, obsessive compulsive disorder (OCD), post-traumatic stress disorder (PTSD) and schizophrenia, allowing us to compare both within and across disorders. We limit our review to studies with an N of at least 20 participants that reported quantifiable results in at least one frequency band. Our intention was not to perform a full-scale meta-analysis but rather a comprehensive review of the state of recent literature. To do so we conducted a search of PubMed^5^ in May 2018 using combinations of the following keywords in the title or abstract: quantitative OR qEEG OR ongoing/on-going OR spontaneous OR resting/rest, combined with EEG and the key terms for each of the disorders of interest. Only studies that examined EEG spectral differences in at least one frequency band (exclusively or alongside other EEG metrics) between a clinical and a control group were included. Studies whose research focus was on other aspects of mental health or cognition, or whose analysis focused exclusively on other EEG metrics (e.g., asymmetry, coherence, microstates, entropy etc.) were excluded. No study was excluded due to methodological limitations, but rather because it missed the proposed research topic. This enabled a comprehensive review of the variability of experimental and clinical parameters across the published literature, rather than restricting it to a particular subset of studies.

As a next step, various methodological parameters were collated including sampling characteristics, EEG recording parameters and power spectrum computation. Sampling characteristics included sample size, demographic data (age, gender), medication status and diagnostic screening method. Key EEG parameters (where available) included referencing style, and recording length, and power spectrum computation included FFT method (windowing function, overlap, epoch length), frequency bands (and frequency window) and whether absolute and/or relative power differences were analyzed within each band. We then noted any reported significant difference (increase or decrease) or lack of significant difference in power/amplitude across each spectral band (delta, theta, alpha, beta, gamma where analyzed) for each study. In addition, to standardize across studies, frequency bands which had been split into sub-bands (e.g., beta1/beta2) were collapsed for all analyses, and where results differed across sub bands (e.g., beta1 showed significance, beta2 showed no significance) we considered the significant finding as the primary result. In addition, in one study (Hong et al., 2012) the theta and alpha bands were collapsed together and in this instance we allocated the result to both bands individually.

Where reported in text or figures, the magnitude of change was also calculated (as a % increase or decrease). Any reported correlations between individual spectral bands and clinical symptoms were also recorded when reported. All collected data were consolidated in a spreadsheet for review and analysis.

### Consistency and Reliability Scores

To determine the dominant result for each band within each disorder group and recording condition we first identified the most frequently occurring (i.e., dominant) result (significant increase, significant decrease or no significant difference). For example, for ADHD in children in the eyes closed condition there were 13 studies reporting a significant increase in the absolute power of the delta band, one study reporting no difference and three studies reporting a significant decrease. In this case the dominant result is a significant increase. When the number of studies showing either a significant increase or decrease was the same as the number showing no difference, the dominant result was considered no difference. When an equal number of studies showed an increase and a decrease (and the number was higher than those showing no significant difference) the result was marked as “opposing.”

We then created a consistency score computed as the ratio of the number of studies reporting the dominant result (e.g., no significant difference) to the number of studies reporting a different result (e.g., significant increase or decrease); essentially how much more frequently the dominant result was reported in the literature compared to some other result. When all studies agreed (i.e., the divisor was zero) we used the number of studies as the consistency score. When the studies were evenly divided between any two results, we computed the consistency score as 1.

We next created a validation score by first computing the average N for the studies showing the dominant result in each band and multiplying this by the number of studies showing the dominant result. We then averaged these values across all the bands (excluding the gamma band which was sparsely reported). The validation score is therefore an indication of the size of the population from which the dominant result was obtained.

## Results

A total of 184 publications published between 1993 and 2018, found using the above search criteria in PubMed, matched our inclusion criteria. A detailed list of studies with key study parameters can be found in Supplementary Table S1. while trends in results are summarized below.

### Overview of Studies

#### Sample Characteristics

**Figure 1:**
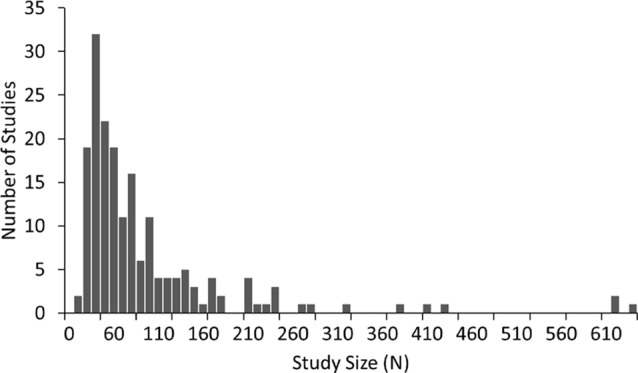
Distribution of sample size across 184 studies in this review. Sample size includes both patients and controls. Median sample size was 60. One study, with a sample size of 1,344, was beyond the scale of this graph.

A summary of the number of studies across disorders and their corresponding sample characteristics is shown in Table 1. Some disorders such as ADHD and schizophrenia were widely studied (65 and 37 articles, respectively) while others such as depression and autism were also popular, though less so (18 and 16 studies respectively). In contrast, some disorders such as bipolar, generalized anxiety and panic disorder were very poorly represented in the literature (six or fewer studies each). The median sample size across the studies was 60, with roughly equal numbers of patients and controls in the majority of studies. Seventy-three percent of studies had sample sizes less than 100 whilst only 10 studies (Clarke et al., 2001d; Wuebben and Winterer, 2001; Rangaswamy et al., 2002, 2006; Magee et al., 2005; McFarlane et al., 2005; Grin-Yatsenko et al., 2009; Kam et al., 2013; Narayanan et al., 2014; Arns et al., 2015) had sample sizes greater than 250 and only one of these (Arns et al., 2015) had a sample size greater than 1,000 (*N* = 1,344; depression; Figure 1). Participants were generally adults with an average age between 30 and 40 except for ADHD and autism where studies largely focused on children and the average age ranged from 5 to 11 years old. Furthermore, samples were typically skewed towards male subjects (64%).

**Table 1:** Overview of studies.

	No. of studies*	Median N	% Controls	Average age (years)	% Females	% Eyes closed^$^
ADHD (children)	56**^#^**	76	45	11	25	75
ADHD (adults)	14**^#^**	55.5	50	33	43	54
Schizophrenia	37	63	54	31	33	92
ASD/Autism^&^	16	56	52	8.5	21	33
Depression	18	55	44	39	57	86
OCD	10	61.5	49	32	56	100
PTSD	13	74	50	40	37	67
Addiction	16	45	49	33	30	88
Panic disorder	4	79	44	35	69	50
Bipolar disorder	6	99.5	55	30	55	50
Anxiety	3	50	50	31	76	50

*Includes five joint studies: (1) depression/post-traumatic stress disorder (PTSD); (2) schizophrenia/bipolar; and (3) schizophrenia/depression. ^$^Includes studies which had only eyes closed as well as studies which included both eyes closed and eyes open. ^&^Excludes the one adult ASD/autism study. ^#^Includes five studies with both children and adult participant groups.

Each study compared a group with a diagnosed disorder to a control group. The majority of studies report on only one disorder, although a minority compare two disorders, e.g., bipolar disorder and schizophrenia (Clementz et al., 1994; Kam et al., 2013; Narayanan et al., 2014), depression and schizophrenia (Begić et al., 2011), depression and PTSD (Kemp et al., 2010), alcohol and internet addiction (Son et al., 2015). In each case, the disorder group(s) were determined using common psychiatric questionnaires as described in Supplementary Table S2. In the majority of studies (70%), patients were unmedicated which was defined as being medication naive or having abstained from taking medication for a predefined period of time (ranging from 12 h to 3 months).

#### Reported Metrics

The majority of studies reported resting state EEG with eyes closed recordings (66% of studies). However, a minority of studies reported results for eyes open (19% of studies) or both eyes open and closed (15%), analyzed either combined or separately. While some studies reported all frequency bands, many were selective in reporting only one or two bands. Across the studies, the alpha and theta bands were the most frequently reported (in 85/84% of studies), followed by beta (80%) and delta (70%). Gamma is the least frequently reported (only 18% of studies). Given this pattern of reporting, it is sometimes unclear when a study reported on only one or two bands, whether it was because the other bands were not analyzed, or whether they were excluded on account of negative or null results. Underreporting of negative or null results may therefore bias this review towards the positive results. It is also important to note that while most studies followed a typical definition for the theta and alpha bands, there was wide variation in the definitions of other bands (see “Methodological Challenges and Limitations” section).

For each band, studies most often reported differences in the absolute power between control and disorder groups (61% of studies). Some of these studies additionally reported relative power (28%) while a few reported differences in relative power only (10%). Relative power is typically calculated by computing the power of each given band divided by the sum of power across all bands. Surprisingly, 29% of studies did not explicitly indicate the method of reporting and required some inference. Where a study did not mention whether it reported absolute or relative data, it was generally assumed that it was absolute in the absence of any evidence to the contrary. Most studies reported aggregated results for broad cortical or source localized regions (60%) while others reported results for individual channels (32%). A small minority provided results aggregated across all recorded channels (8%). Given these differences in reporting we computed the magnitude of difference between the control and disorder groups as percentages, where the information was available (in 40% of cases), averaging across broad regions in all studies. Where there was a regional split between increases and decreases across the scalp (e.g., frontal increases and posterior decreases) the regional magnitudes were allocated to their respective increase and decrease groupings (rather than being averaged together). Finally, a proportion of studies (27%) additionally reported correlations (significant or non-significant) between individual bands and disorder severity.

It is also important to note that although some of the studies reported here exclusively focused on the analysis of the power spectrum, many of them additionally reported on other metrics including coherence analysis and asymmetries which are not reported here.

### Aggregate Trends Across Frequency Bands and Disorders

A trend analysis was performed for both absolute and relative power differences reported between the disorder and control group in each band (definitional and other methodological differences notwithstanding) for each disorder. To standardize across studies, we collapsed across any bands which had been split into sub-bands (e.g., beta1/beta2). Where results differed across bands (e.g., beta1 showed significance, beta2 showed no significance) we considered the significant result as the primary result. Furthermore, results are shown separately for the eyes closed and eyes open conditions. A small number of studies that combined eyes open and closed (for opioid addiction, depression, panic disorder, anxiety) are excluded from the trend analysis but displayed in the tables for completeness. In addition, in the minority of cases where there was only a single study condition (i.e., eye open/closed, absolute/relative) for a particular disorder, the study was not included in the summary table or trend analysis.

#### Dominant Results Across Disorders

The number of studies reporting either a significant increase, a significant decrease, or no significant difference in the power in each of the frequency bands relative to control for each disorder are shown in Supplementary Tables S3, S4 for absolute and relative power respectively. The dominant result for each band within each disorder group and recording condition (significant increase, significant decrease or no significant difference) was determined based on the result reported by the greatest number of studies as described in the methods section “Consistency and Reliability Scores.”

**Figure 2:**
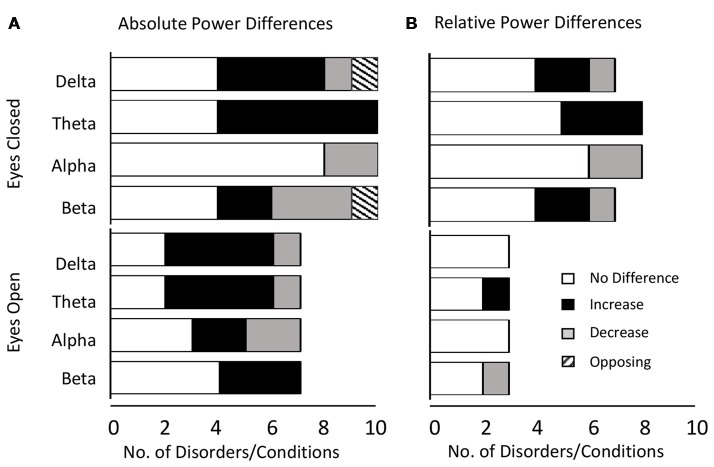
Dominant result aggregated across all disorders and bands. **(A)** Number of disorders with no difference in absolute power relative to controls (white), an increase (black), a decrease (gray) or opposing results (hashed) for eyes closed (top) and eyes open (bottom) conditions. Increases are more common for lower frequency bands (delta and theta) whilst decreases or no significant difference are more common for higher frequency bands (alpha and beta). **(B)** Same as **(A)** for relative power. Legends and axis labels are common.

Altogether we found that the most common result across all disorders and bands combined was an absence of any significant difference in both the eyes closed (53% absolute power, 63% relative power) and relative eyes open conditions (83%), whilst there were similar levels of significant increase (46%) and no significant difference (39%) for absolute eyes open. The dominant results for each band aggregated across all distinct disorders and conditions are shown in Figure 2 for absolute power (Figure 2A) and relative power (Figure 2B).

When restricting our view to the smaller proportion of disorders/conditions where the dominant result was a significant increase or decrease, the general pattern that emerged was that increases dominated in the lower frequency delta and theta bands (86% for absolute and relative power) while decreases dominated in the alpha band (67% absolute, 100% relative). In contrast decreases were roughly as likely as increases in the beta band depending on the condition (37.5% absolute, 50% relative). The gamma band was excluded from analysis due to the small number of studies, although here again, decreases were more common.

**Figure 3:**
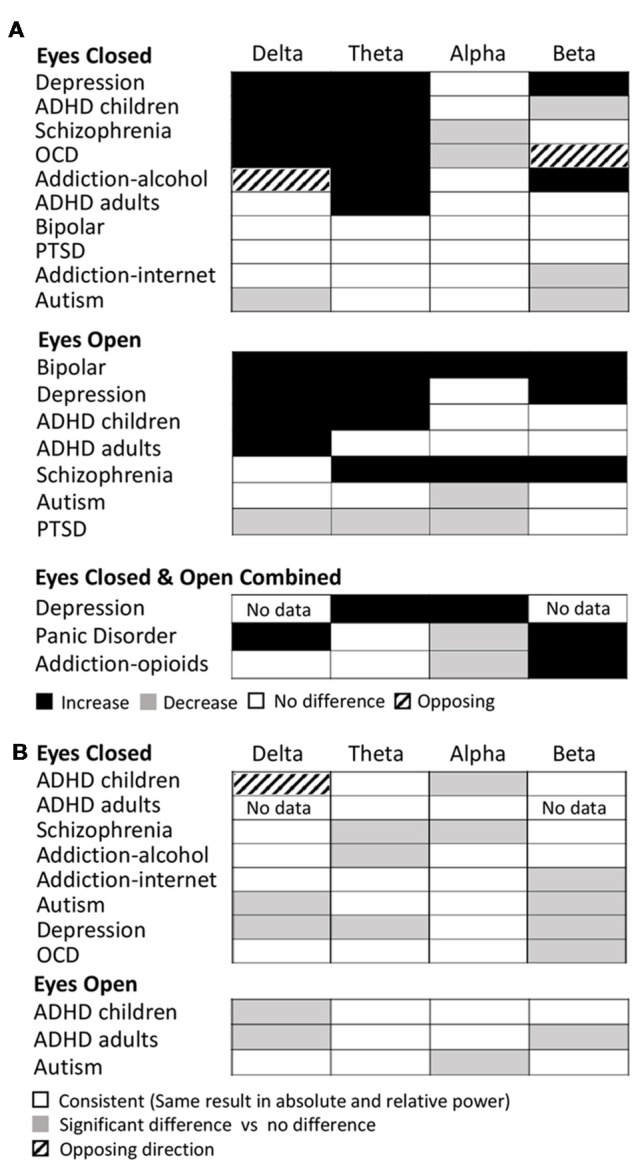
Dominant results for each individual disorder and band. **(A)** Differences in absolute power for each disorder (relative to control) for eyes closed condition (top), eyes open (middle) and eyes open and closed combined (bottom). White boxes indicate no change, black indicates an increase, and gray indicates a decrease. Opposing results are shown by hashed boxes. **(B)** Difference in results between absolute power and relative power for the same disorders. White indicates no difference, gray indicates a significant increase or decrease in one but no significant difference in the other, while a hashed box indicates opposite results.

Examining this general effect at the level of the individual disorder types, the results showed that there was an increase in absolute power for both delta and theta in the eyes closed condition for ADHD (in children), schizophrenia, OCD and depression, while ADHD (in adults) and alcohol addiction showed an increase only in the theta band (Figure 3A). In the eyes open condition, an increase was dominant in both delta and theta for depression, ADHD (in children) and bipolar disorder but only in the delta band for ADHD (adults) and only in the theta band for schizophrenia. However, even across those disorders where an increase dominated these bands, there were nonetheless a minority of studies reporting the opposite effect (e.g., three studies of ADHD in children, Dupuy et al., 2014a; Giertuga et al., 2017; Shephard et al., 2018, two studies of schizophrenia Pascual-Marqui et al., 1999; Knyazeva et al., 2008 and one of OCD, Bucci et al., 2004). The only cases in the lower frequency bands where the dominant result was a decrease, rather than an increase, was in the delta band for autism (eyes closed, Coben et al., 2008) and in the theta and delta bands for PTSD/early life stress (eyes open, McFarlane et al., 2005; Veltmeyer et al., 2006).

Significant decreases in absolute power were dominant in the alpha band for schizophrenia and OCD (eyes closed ), autism and PTSD (eyes open), and in the beta band for ADHD (children), autism and internet addiction (all eyes closed; Figure 3A). In contrast, significant increases were dominant in a handful of disorders, most frequently when participants had their eyes open, including depression (beta, eyes open and closed), bipolar (alpha and beta, eyes open), schizophrenia (alpha and beta, eyes open) and alcohol addiction (beta, eyes closed ).

In two cases (OCD, eyes closed beta band and alcohol addiction, eyes closed delta band) there was no dominant result but rather an equal number of studies showing increases and decreases. These are shown as hash marked in Figure 3A.

In Figure 3B we show the differences between the dominant result for absolute and relative power differences. Overall the dominant result for relative power was the same as for absolute power in 62% of experimental comparisons (white squares) where each comparison is one band within one disorder and condition. Results were most similar across relative and absolute for the theta band (73% of disorders/conditions). Cases where there was a significant difference in one method but not the other are indicated as gray (36% of disorders/conditions), which, when examined in more detail, was the case for 50% of the disorders and conditions in the beta band and 40% in the delta band. There was a greater proportion of disorders/conditions with no significant difference for relative power compared to absolute power. This was particularly true for the delta band. The only case where the dominant result was diametrically opposed for absolute power vs. relative power was in the delta band for ADHD in children (eyes closed) where there was an increase in the absolute power and decrease in the relative power (Figure 3B, hashed box).

Given the overall pattern of a greater likelihood of increases in the lower frequencies and no change or decreases in higher frequencies, it is important to note that, with the exception of ADHD, the same disorders that were dominated by increases in theta were not the ones dominated by decreases in beta. However, the overall trend across disorders would be for a decreased theta/beta ratio either due to an increase in theta and decrease in beta, an increase in theta and no change in beta, or no change in theta and a decrease in beta.

#### Consistency of Results

**Figure 4:**
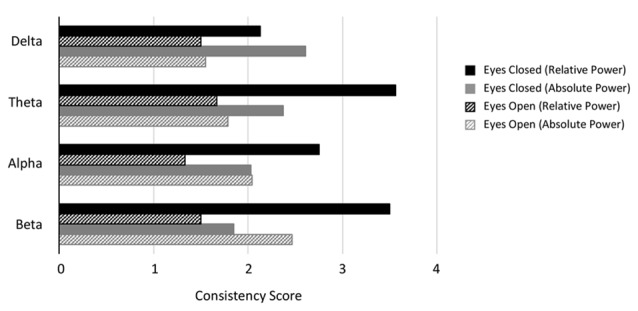
Consistency scores aggregated across disorders for each band and condition. Consistency scores (frequency of dominant result relative to other results) were between 2 and 3 for absolute power in the eyes closed condition for all bands (gray bars), between 2 and 4 for relative power eyes closed (black bars) and typically between 1 and 2 for eyes open (absolute and relative, gray and black hashed bars, except beta eyes open absolute power).

**Figure 5:**
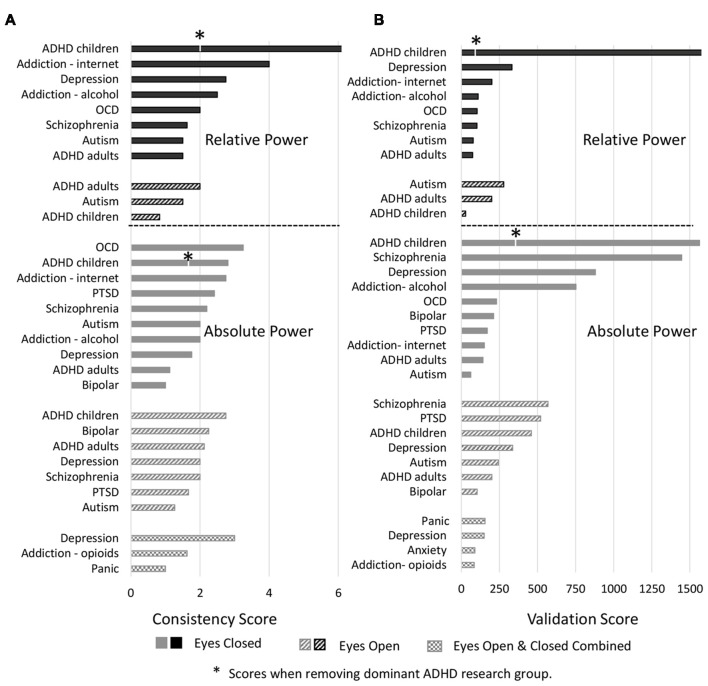
Consistency and validation scores by disorders. **(A)** Consistency scores for each disorder for relative power (top) with eyes closed (black bars) and eyes open (black hashed bars) and absolute power (bottom) with eyes closed (gray bars), eyes open (gray hashed bars) and eyes closed and open combined (hatched bars). Asterisk marks for attention deficit-hyperactivity disorder (ADHD) indicate consistency scores when the dominant research group is excluded. **(B)** Validation scores for each disorder. Order and legend are as in **(A)**. Validation score for ADHD in children, relative power with eyes closed goes beyond the scale of this graph (2,516).

We next report analysis of consistency of the results for those disorders/conditions where there were at least two studies reporting on any particular band (Figures 4, 5). Consistency scores were calculated as described in methods section “Consistency and Reliability Scores” and can be read as how much more frequently the dominant result occurred in the literature compared to any other result.

Figure 4 shows the average consistency scores across all disorders for each band for the eyes closed (solid bars) and eyes open conditions (hashed bars), for both absolute and relative power (gray and black bars respectively). Overall, the highest consistency score, aggregated across all disorders and bands was for relative power with eyes closed (3.0) followed by absolute power with eyes closed (2.2). Eyes open had lower consistency with 2.0 and 1.5 for absolute and relative power respectively. When calculated separately for each band, a similar pattern was observed, though scores were slightly lower overall for delta and alpha. Taken together this suggests that eyes open is a much more variable condition and that relative power estimates are more reliable.

Analysis of individual disorders/conditions, aggregated across bands (Figure 5A), revealed that the highest consistency score was for relative power comparisons of controls to ADHD in children with eyes closed (7.0) followed by internet addiction with eyes closed (4). The highest consistency scores for absolute power with eyes closed was for OCD (3.3), internet addiction (2.8) and ADHD in children (2.8). Autism and ADHD in adults had generally the lowest consistency across all conditions. It is significant, however, that the literature for two disorders with the highest consistency scores, ADHD in children and internet addiction, were each dominated by a single research group (47% of the articles for ADHD, 100% for internet addiction) which was not the case for other disorders with multiple studies. This has the advantage of a consistent methodology but also risks bias. We thus point out the consistency score for ADHD in children when the dominant group is removed with an asterisk (Figure 5A).

We next report validation scores, computed as described in methods sections “Consistency and Reliability Scores,” that are essentially the total number (N) of study participants across all the studies reporting the dominant result (Figure 5B). ADHD in children with eyes closed had the highest number of studies showing the dominant result (8–25 per band) and with an average N of 129 the validation scores were the highest with 2,516 for relative power (beyond the scale of the graph) and 1,563 for absolute power. We note however that this more than halves for relative power when the dominant research group is excluded. Also high was schizophrenia with 1,446 for absolute power followed by depression (absolute, eyes closed) with 880. Nineteen percentage of disorders/conditions had scores less than 100 and 47% had less than 200 indicating that they involved few studies and participants and therefore cannot be considered to be sufficiently validated results.

#### Magnitude of Results

We next considered the reported magnitudes of difference (in %) for absolute and relative power, averaged across only those studies where a significant difference was reported, and where accurate information was available in the text, tables or figures of the publication (shown in detail in Supplementary Table S5). On average, 40% of study comparisons reported magnitude data, although this varied across disorder types and ranged from 68%, 67% and 58% for ADHD (adults), ASD/Autism and bipolar disorder respectively at the upper end, through to 26% for ADHD (children) and 29% for OCD at the lower end (in addition, no anxiety studies identified for this review included magnitude data).

**Figure 6:**
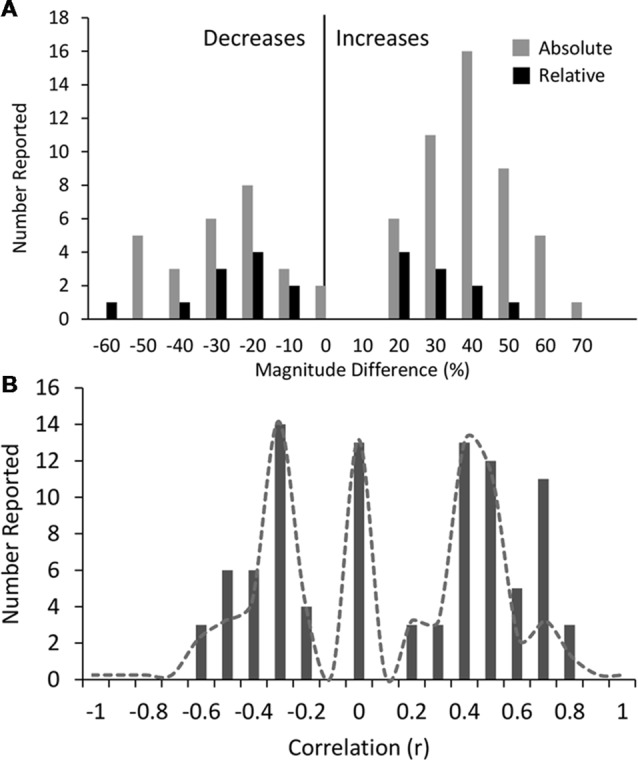
Histograms of magnitudes of differences and correlations. **(A)** All reported magnitudes for differences between disorder groups and controls across all bands for absolute (gray bars) and relative power (black bars). Increases are shown as positive and decreases are negative. Reported increases for absolute power outnumbered reported decreases, although were similar in magnitude (35% and 34% respectively). Relative power was relatively symmetric with average magnitude increases of 22% and decreases of 31%. **(B)** Histogram of all reported correlations of band increases or decreases with symptom severity scores (all results were included even if not the dominant result). Reports of “no significant correlation” are shown as 0. Positive and negative correlations were typically between 0.2 and 0.5 although positive correlations were higher on average. High correlations (>0.6) were only found in two small studies (*N* < 40). Dotted line shows histogram excluding these two studies.

Across all disorders/conditions, the reported magnitude of difference (mean ± SD) was 34 ± 13% for absolute power and 26 ± 14% for relative power, irrespective of whether the reported result was the dominant one or not. The distribution of magnitudes is shown in Figure 6A. Overall the magnitude of increases (vs. decreases) were higher on average for absolute power (gray bars) but not relative power (black bars). Given that magnitude data was not consistently reported across bands and conditions, no disorder or band specific trend can be reliably inferred. We therefore do not report any trends. However, we do note that reported magnitudes were highest for schizophrenia, depression and bipolar disorder (~44% on average for eyes closed and ~48% on average for eyes open across all bands) and lowest for opioid, internet addiction, ADHD in children with eyes open and PTSD with eyes open (all 21%–22%). Overall magnitudes were also highest for the alpha band, particularly for decreases reported with eyes closed (46% on average) while other bands were similarly lower.

We note that in many cases where different studies reported opposing results, the magnitudes reported were not very different. For example, although the dominant result for schizophrenia was a decrease in alpha (on average 58%), those studies that reported an increase in alpha (Hong et al., 2012; Kim et al., 2015) reported a similar magnitude (64%).

#### Correlation With Disorder Severity

We also looked at reported correlations between individual bands and disorder severity, as rated by the clinical diagnosis and symptom questionnaire (Supplementary Table S6). Twenty-seven percent of studies reported multiple such correlations for different bands and brain regions. We included all reported correlations regardless of the specific brain region or band or symptom subset for which the correlation was reported. The distribution of these correlations is shown in Figure 6B. The correlations generally ranged from 0.2 to 0.5 with an average around 0.4 (positive or negative) while a fraction of instances reported no significant correlation (shown as 0). It is highly likely that the nonsignificant correlations are underreported. Higher correlations of 0.6–0.8 were reported in some studies showing a second peak in the distribution. However, these were disproportionately from two studies (Pogarell et al., 2006; Roh et al., 2015) with a very small number of participants (less than 40). When these were excluded, the peak at 0.7 was much reduced (shown by the dotted line). Further, there were no notable differences in the correlations for any individual disorder or band. In addition, some studies reported regression coefficients rather than correlations which were generally lower (between 0.2 and 0.3) and are not included in the distribution. Thus, as an overall conclusion, it appears that correlations of band power to symptom scores are generally weak and not specific to any band or disorder.

We note that some studies included correlations to other factors such as a particular task performance, demographic variables or age of onset that are not reported here. In addition, a handful of studies performed other types of diagnosis classification modeling to distinguish and predict differences between the two study groups (Kim et al., 2015: schizophrenia; Knott et al., 2001b; Deldin and Chiu, 2005: depression; Chan and Leung, 2006; Chan et al., 2007; Sheikhani et al., 2012: autism; Kim et al., 2017: internet addiction; Ogrim et al., 2012; Buyck and Wiersema, 2014a; Poil et al., 2014; Markovska-Simoska and Pop-Jordanova, 2017: ADHD). Again, these are not reported here.

### Individual Psychiatric Disorders

#### ADHD

This review identified 65 ADHD studies with a median sample size of 76 (children) and 55.5 (adults; range 23–378). Of these, 56 studied children and adolescents (average age of 11 years; Kuperman et al., 1996; Clarke et al., 1998, 2001a,b,c,d, 2002a,b,c,d,e,f, 2003, 2006, 2007, 2008b, 2011, 2013, 2016; Bresnahan et al., 1999; Swartwood et al., 2003; Hermens et al., 2005a,b,c; Magee et al., 2005; Hobbs et al., 2007; Fonseca et al., 2008, 2013; Barry et al., 2009a,b, 2010; Sohn et al., 2010; Dupuy et al., 2011, 2013, 2014a,b; Lansbergen et al., 2011; Ogrim et al., 2012; Shi et al., 2012; Liechti et al., 2013; Buyck and Wiersema, 2014a,b, 2015; Poil et al., 2014; Tye et al., 2014; Kitsune et al., 2015; Roh et al., 2015; Kamida et al., 2016; Kim et al., 2016; Thomas and Viljoen, 2016; Giertuga et al., 2017; Jarrett et al., 2017; Markovska-Simoska and Pop-Jordanova, 2017; Park et al., 2017; Rommel et al., 2017; Shephard et al., 2018) and 14 studied adults (average age of 33 years; Bresnahan et al., 1999, 2006; Bresnahan and Barry, 2002; Hermens et al., 2004; Clarke et al., 2008a; Koehler et al., 2009; van Dongen-Boomsma et al., 2010; Woltering et al., 2012; Liechti et al., 2013; Buyck and Wiersema, 2014a; Poil et al., 2014; Rommel et al., 2016; Markovska-Simoska and Pop-Jordanova, 2017; Tombor et al., 2018). Five of these studies included both adults and children as participant groups (Bresnahan et al., 1999; Liechti et al., 2013; Buyck and Wiersema, 2014a; Poil et al., 2014; Markovska-Simoska and Pop-Jordanova, 2017). Above and beyond DSM or ICD in these studies, diagnosis for ADHD was most typically performed using the Conners’ Parent Rating Scale (CPRS; Conners et al., 1998), the Child Behavior Checklist (CBCL; Achenbach and Rescorla, 2001), the Wender Utah Rating Scale (WURS; Ward et al., 1993), Conners’ Adult ADHD Rating Scales (CAARS; Conners and Sparrow, 1999) and Barkley’s Semi-structured Interview for Adults with ADHD (Barkley, 2011; Supplementary Table S2).

**Figure 7:**
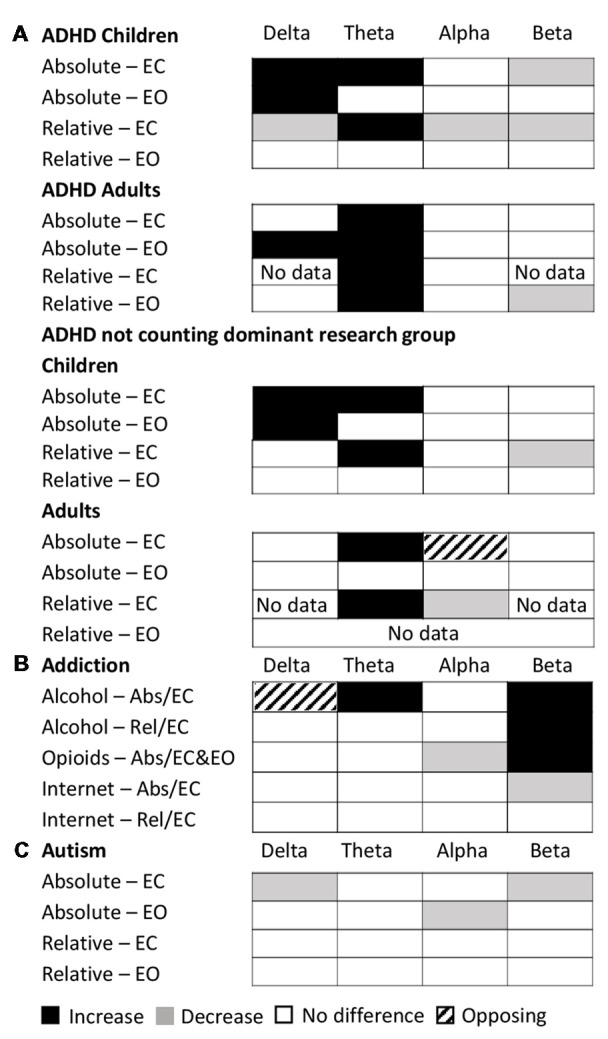
Dominant results across conditions for specific disorders. **(A)** Dominant result for each band for ADHD for each of various conditions and age groups for both relative (rel) and absolute (abs) power and for eyes closed (EC) and eyes open (EO), including all studies (top) and excluding the dominant research group (bottom). White boxes indicate no change, black indicates an increase, and gray indicates a decrease. Opposing results are shown by hashed boxes. **(B)** Dominant result for each band for each type of addiction and condition. Legend as in **(A)**. **(C)** Dominant result for each band for autism for each condition. Legend as in **(A)**.

The dominant results for all age groups and conditions are shown in Figure 7A. Overall as described above, the results for relative power in ADHD in children with eyes closed had very high consistency (7.0) and validation (2,516) scores. Absolute power for eyes closed was still reliable but less so, with a consistency score of 2.8 and validation score of 1,563. The eyes open condition had a consistency score of 2.8 and validation score of 460. On the other hand, studies in adults were substantially less consistent (consistency scores of 2.1 for eyes open and 1.1 for eyes closed for absolute power, and 2 for eyes open and 1.5 for eyes closed for relative power) and poorly validated (75–201 depending on condition). However, given that nearly half the studies reported for ADHD, particularly for children, came from a single research group (31 out of 65), we also show here the results when excluding this group (Figure 7A, bottom). As can be seen, in the absence of this group the dominant result for both delta and theta increases for children remains, as does the decrease in beta activity for relative eyes closed for children, but there is a greater number of “no-significant difference” results overall for children. For adults, theta increases persist for absolute and relative eyes closed, but differences are observed for the other conditions and bands.

The following nine ADHD studies in children (absolute power; eyes closed) failed to see the decrease in the beta band (Clarke et al., 2001d; Hobbs et al., 2007; Fonseca et al., 2008; Liechti et al., 2013; Buyck and Wiersema, 2014b, 2015; Dupuy et al., 2014a; Kamida et al., 2016; Kim et al., 2016) whilst three further ADHD studies reported a decrease in beta in posterior regions but an increase in frontal regions (Clarke et al., 2002a, 2011, Hermens et al., 2005a). These inconsistencies could be due to differences in the methodological approach (see below) or demographic or clinical differences within the participant groups.

Overall, considering all studies, there is a reasonable confidence in the general trend reported in the results for children but not adults, particularly for relative power eyes closed. However, in all cases the magnitudes of difference were modest (~28% for both absolute and relative power) and correlations with symptom severity were typically in the order of 0.3–0.4 for all bands (with the exception of Roh et al., 2015) which reported correlations between 0.6 and 0.7 across all bands and brain regions for a very small sample set). This indicates that overall results pertaining to frequency bands are not sufficiently discriminatory nor predictive of symptoms.

Given the reliable increase in theta and concomitant decrease in beta reported in children under the eyes closed condition, the theta/beta ratio had been proposed and approved by the FDA as a diagnostic biomarker for ADHD. However, the lack of consistency in adults suggests that these findings are likely age dependent and can perhaps not be extrapolated beyond the narrow age group studied. Furthermore, the general pattern of either an increase in theta or a decrease in beta is shared by a number of other disorders including OCD, schizophrenia and internet addiction suggesting that a reduced theta/beta ratio is a general marker for shared symptoms across a number of disorders rather than specific to the diagnosis of ADHD. However, we acknowledge that there are some studies which specifically examine the theta/beta ratio, without reporting results from individual spectral bands and therefore did not meet the inclusion criteria for this review (e.g., see Arns et al., 2013). Reported results relating to the theta/beta ratio may therefore be underreported in this review. For this we point the reader to a number of recent reviews and meta-analyses of EEG and ADHD, which have tried to detangle the pattern of EEG frequency band changes across studies (Barry et al., 2003; Snyder and Hall, 2006; Loo and Makeig, 2012).

#### Schizophrenia

A number of resting-state EEG studies have been conducted on patients with schizophrenia (vs. healthy controls) most often with eyes closed (although see Venables et al., 2009; Hanslmayr et al., 2013; Narayanan et al., 2014 for three eyes open studies). In total, 37 schizophrenia studies were identified for this review (Clementz et al., 1994; Sponheim et al., 1994; Wada et al., 1994; Omori et al., 1995; Pascual-Marqui et al., 1999; Begić et al., 2000; Harris et al., 2001; Knott et al., 2001a; Wuebben and Winterer, 2001; Mientus et al., 2002; Veiga et al., 2003; Kirino, 2004; Harris et al., 2006; Kirino, 2007; Knyazeva et al., 2008; Tislerova et al., 2008; John et al., 2009; Venables et al., 2009; Bandyopadhyaya et al., 2011; Begić et al., 2011; Itoh et al., 2011; Schug et al., 2011; Hong et al., 2012; Hanslmayr et al., 2013; Kam et al., 2013; Narayanan et al., 2014; Ranlund et al., 2014; Tikka et al., 2014; Andreou et al., 2015; Garakh et al., 2015; Goldstein et al., 2015; Kim et al., 2015; Mitra et al., 2015; Shreekantiah Umesh et al., 2016; Mitra et al., 2017; Moeini et al., 2017; Baradits et al., 2018). The median sample size was 63 (range 26–425), with the average age of participants being 31 years old. As well as more conventional DSM/ICD measures, schizophrenia diagnosis and severity was typically assessed using the Positive and Negative Syndrome Scale (PANSS; Kay et al., 1987) and the Brief Psychiatric Rating Scale (BPRS; Overall and Gorham, 1962).

Schizophrenia showed consistent and reliable increases in the absolute delta and theta band power and decreases in the absolute alpha band power compared to controls with eyes closed (consistency scores of 2.2, reliability score 1,446). Furthermore, these differences were higher in magnitude relative to differences reported for other disorders (average theta increase of 50%, alpha decrease of 58%). The net result would be a higher theta/beta ratio compared to controls, very similar to ADHD in children. However, the three eyes open studies (Venables et al., 2009; Hanslmayr et al., 2013; Narayanan et al., 2014) showed a completely different pattern—an increase in theta, alpha and beta activity. Regional differences were also observed in a handful of studies in the delta (Begić et al., 2000) and alpha (Omori et al., 1995; Kim et al., 2015) bands where there was a frontal-posterior split with frontal increases and posterior decreases for alpha and the opposite pattern in the delta band. In addition, we found only three studies (Kirino, 2004, 2007; John et al., 2009) which measured relative changes in spectral power, the majority of which showed non-significant differences across all bands (although see John et al., 2009).

#### Depression

Eighteen depression studies were identified for this review (Kwon et al., 1996; Bruder et al., 1997; Bell et al., 1998; Debener et al., 2000; Knott et al., 2001b; Pizzagalli et al., 2002; Deldin and Chiu, 2005; Morgan et al., 2005; Bruder et al., 2008; Korb et al., 2008; Price et al., 2008; Grin-Yatsenko et al., 2009; Kemp et al., 2010; Begić et al., 2011; Jaworska et al., 2012; Cook et al., 2014; Arns et al., 2015; Slobodskoy-Plusnin, 2018). The median sample size was 55 (range 21–1344) with the average age of participants being 39 years old. Beyond more conventional DSM/ICD measures, depression diagnosis and severity was most typically measured using the Hamilton Rating Scale for Depression (HAM-D; Hamilton, 1960).

The dominant result for depression was an increase in the absolute power in both theta and beta bands for both eyes open and eyes closed conditions (eyes closed consistency 1.8, validation 880; eyes open consistency 2.0, validation 337) with average magnitudes of 48%. However, these increases were no longer visible when considering relative power where most studies failed to find any significant differences across any band (Knott et al., 2001b; Morgan et al., 2005; Korb et al., 2008; Cook et al., 2014). The largest study (Arns et al., 2015) consisting of 1,344 participants showed increases in theta power across frontal regions of the brain using the eLORETA source localized signal which is methodologically different from most other depression studies identified for this review which perform their analysis in electrode space.

#### Addiction

Here, we focus on three major types of addiction: opioids, alcohol and the internet and identified 16 addiction studies in this review. The median sample size was 45 (range 28–614), with the average age of participants being 33 years old. Beyond more conventional DSM/ICD measures, diagnosis and severity of internet addiction was most typically performed using the Young’s Internet Addiction Test (IAT; Young, 1998), whilst alcohol and opioid addiction were assessed using a variable set of questionnaires depending on the study.

Surprisingly, despite the enormous attention to opioid addiction by both media and government, particularly in the United States, only four resting-state EEG studies (Wang et al., 2015b, 2016; Motlagh et al., 2017; Zhao et al., 2017) were identified for this review based on our inclusion criteria (for other reviews, see Wang et al., 2015a; Ieong and Yuan, 2017). In addition, nine alcohol addiction (Günther et al., 1997; Bauer, 2001; Rangaswamy et al., 2002, 2006; Saletu-Zyhlarz et al., 2004; Fein and Allen, 2006; Andrew and Fein, 2010; Son et al., 2015; Herrera-Díaz et al., 2016) and four internet addiction (Choi et al., 2013; Lee et al., 2014; Son et al., 2015; Kim et al., 2017) studies were identified for this review (includes one publication which examined both alcohol and internet addiction in the same study). It is important to acknowledge that addiction is a heterogeneous label encompassing multiple “types” of addictive disorder, and that the similarities and differences in the underlying etiologies between substance addiction and internet addiction are still not well defined. However, with the recent inclusion of gaming addiction in the 11th Revision of the International Classification of Diseases (ICD-11)^6^, we have included internet addiction alongside substance addiction disorders for interest and comparison.

The dominant result across all addictions and conditions was one of no significant difference in all bands of the power spectrum except beta which showed an increase for opioid and alcohol addiction and a decrease for internet addiction (Figure 7B). In addition, there was an increase in theta power for alcohol addiction, and a decrease in alpha power for opioid addiction. Even where significant differences were reported, the magnitudes were small (15%–27%). While internet addiction had a high consistency score of 4, all four studies came from the same research group. Alcohol addiction had a consistency score of 2.25 while the studies for opioid addiction were too few in each condition to calculate a consistency score. Overall, across all addictions, the validation scores ranged (from 35 to 753 depending on addiction type and condition). Given the small number of studies and high methodological variability, dependable conclusions cannot yet be drawn. However, as it stands, other than for the beta band, the power spectrum appears essentially unaffected in any consistent and reliable way by addiction.

#### OCD

Ten OCD studies were identified (all eyes closed) for this review (Molina et al., 1995; Tot et al., 2002; Karadag et al., 2003; Bucci et al., 2004; Pogarell et al., 2006; Velikova et al., 2010; Kopřivová et al., 2011, 2013; Olbrich et al., 2013; Kamaradova et al., 2016) with an median sample size of 61.5 (range 26–100). The average age of participants was 32 years old. Five of these studies analyzed spectral power in the source localized signal (Velikova et al., 2010; Kopřivová et al., 2011, 2013; Olbrich et al., 2013; Kamaradova et al., 2016), however source localized results were not substantially different from non-source localized studies. Beyond more conventional DSM/ICD measures, OCD diagnosis and severity was typically performed using the Yale-Brown Obsessive Compulsive Scale (Y-BOCS; Goodman et al., 1989).

Like ADHD (in children) and schizophrenia, the dominant pattern was an increase in the delta and theta bands (average increases of ~27 and 36% for absolute and relative power respectively) and a decrease in the alpha band (average decrease of 41%). Further this pattern had a high consistency score of 3.3 and a validation score of 231 for absolute power. On the other hand, relative power was highly inconsistent (score 2) and poorly validated (score 104).

OCD is often comorbid with other mental disorders and therefore the pattern of EEG frequency band differences is unlikely to reflect changes that are purely attributable to OCD. There may also be overlap in symptoms with ADHD (Abramovitch et al., 2015) and schizophrenia (Cunill et al., 2009).

#### PTSD

Thirteen studies with patients with PTSD (Begić et al., 2001; Jokić-Begić and Begić, 2003; Ehlers et al., 2006; Rabe et al., 2006; Veltmeyer et al., 2006; Falconer et al., 2008; Shankman et al., 2008; Kemp et al., 2010; Todder et al., 2012; Wahbeh and Oken, 2013; Imperatori et al., 2014; Clancy et al., 2017), and with individuals who have suffered significant early life stress (McFarlane et al., 2005), were identified for this review. The median sample size was 74 (range 20–407), with the average age of participants being 40 years old. In addition to conventional DSM/ICD measures, PTSD diagnosis and severity was most typically performed using the Clinician-Administered PTSD Scale (CAPS; Blake et al., 1995).

The majority of eyes closed studies indicate no significant differences in spectral bands between PTSD patients and controls with a reasonable consistency score of 2.4 for absolute power. When differences were reported, they suggest a decrease in all bands in the disorder group for eyes open conditions, and both increases and decreases for eyes closed conditions. However, in most of these studies while “significant” effects are stated, specific numbers pertaining to the magnitude are not reported making it difficult to evaluate.

#### Autism

Seventeen studies with patients with autism or ASD were identified for this review (Dawson et al., 1995; Sutton et al., 2004; Chan and Leung, 2006; Chan et al., 2007; Orekhova et al., 2007; Stroganova et al., 2007; Coben et al., 2008; Burnette et al., 2011; Mathewson et al., 2012; Sheikhani et al., 2012; Tierney et al., 2012; Machado et al., 2015; Maxwell et al., 2015; van Diessen et al., 2015; Jaime et al., 2016; Kozhushko et al., 2018; Lefebvre et al., 2018). These have primarily been conducted with children with the average age of participants (children) being 8.5 years old (but see Mathewson et al., 2012 for an example of a study with adults, and not included in the trend analysis). The median sample size was 56 (range 25–156). Beyond more conventional DSM/ICD measures, autism diagnosis and severity was typically performed using Autism Diagnostic Interview-Revised (ADI-R; Lord et al., 1994), the Autism Diagnostic Observation Schedule (ADOS; Lord et al., 1989) and the Social Communication Questionnaire (SCQ; Rutter and Lord, 2003).

Overall autism showed little or no significant difference in the majority of bands (with the exception of delta and beta eyes closed and alpha eyes open; Figure 7C). However, the results for autism are highly inconsistent (consistency scores all below 2), and no general pattern can be inferred.

#### Other Disorders

Other disorders such as bipolar disorder (Clementz et al., 1994; El-Badri et al., 2001; Başar et al., 2012; Kam et al., 2013; Narayanan et al., 2014; Moeini et al., 2015), anxiety (Sachs et al., 2004; Oathes et al., 2008; Xing et al., 2017) and panic disorder (Knott et al., 1996; Gordeev, 2008; Wise et al., 2011; de Carvalho et al., 2015) are included here for completeness. However generally there was no more than one or two studies for any one condition (eyes closed, eyes open, relative power, absolute power), which was too few for the inference of any trends or for the calculation of consistency scores. Nonetheless we show these results as part of our table with the caveat that they are generally poorly validated.

#### Summary

In summary, differences reported for ADHD in children stood out as being the most consistent and validated, although published results were dominated by a single research group. The trends for schizophrenia could be considered as the next most reliable with a trend similar to ADHD in children. Others such as OCD, depression and internet addiction are moderately reliable while the results for other disorders or conditions are either too sparse or inconsistent to be considered reliable.

### Methodological Challenges and Limitations

One considerable challenge when reviewing the literature is the range of methodologies employed that result in difficulties comparing one study to another. Here, we outline the differences in participant selection, EEG recording and analysis that could impact the results reported in this review.

Several sets of EEG guidelines have been published over the years, including guidelines from the American Clinical Neurophysiological Society^7^ as well as from other published studies (e.g., Pivik et al., 1993; Roach and Mathalon, 2008; Keil et al., 2013; Webb et al., 2015). These discuss the various factors that need to be considered when choosing which EEG parameters to use. For example, Keil et al. (2013), emphasize the multitude of parameters which can influence the transformation of the power spectrum and highlight the importance of noting the parameters that influence the final reported outcome. For example, in relation to the Fourier transform, they state that “Researchers” should indicate the type, size, and overlap of the window functions used,” reminding researchers that “When using commercial software, it is not sufficient to indicate that the spectrum was calculated using a particular software package.” However, when looking across the 184 resting-state EEG studies identified for this review it is apparent that there is very poor compliance to many of these standardization recommendations. For example, several studies in this review, simply state that the data was transformed by FFT without providing any further details of the parameters used. This lack of standards presents a general confound for the field that extends beyond the implications for this particular review.

#### Study Size, Composition and Controls

The sample size of studies varies between *n* = 20 and *n* = 1,344 with three quarters of studies based on less than 100 participants. The median is 60 (Figure 1) with similar numbers of controls and patients in the majority of studies. For most studies the age of participants were adults in the range of 25–45.

Interestingly most of the studies in this review were skewed towards male participants (64% compared to 36% female). This pattern is found for all disorders except for depression, bipolar disorder, panic disorder, anxiety and OCD (where the % of females ranged from 55% to 68%). The largest gender disparity is seen for ADHD (72% M/28% F), schizophrenia (67% M/33% F), autism (78% M/22% F) and addiction (70% M/30% F). In addition, it was more common to study all-male participant groups (20% of studies) compared to all-female participants (4% of studies). In some instances, the ratio of males to females was intentionally designed to reflect the relative proportions of sufferers in the general population, but at other times was a reflection of participant availability, limiting the generalizability of these results, especially towards the female population.

There is substantial variability in the EEG both across and within normal individuals that has been reported in the literature (Haegens et al., 2014) that can relate to various factors from task performance (Arazi et al., 2017a,b) to age (Voytek et al., 2015; Hashemi et al., 2016) and socioeconomic factors (Parameshwaran and Thiagarajan, 2017a,b,c). Furthermore, there is a great deal of intra person variability that can arise both naturally and with ingestion of common substances such as caffeine (Kelly et al., 2008; Foxe et al., 2012; Gonen-Yaacovi et al., 2016) and alcohol (Korucuoglu et al., 2016). Only a handful of studies considered inter person variability, relationship to age or intra person variability in their analysis (e.g., see Debener et al., 2000; Chan and Leung, 2006). One study (autism) which did monitor intrapersonal variability by conducting two testing sessions 3 months apart found that amplitudes of theta, alpha and beta significantly differed for patients (but not controls) between the two sessions, although only alpha, and the theta/beta ratio remained significantly different after correction for familywise errors (Chan and Leung, 2006). In addition, the small sample sizes make it challenging to tease out effects of age and normal individual variability from those related to psychiatric symptoms.

ADHD provides an example of studies focused separately on adults and children. The stark difference between the results of these two groups points to changes over the lifespan and it is conceivable that similar studies in the elderly may produce different results still. Without controlling for normal variability and change across the lifespan, it is difficult to know whether these changes are due to the clinical evolution of ADHD, or reflect independent age-related maturation of the EEG.

#### Clinical Groups and Assessment

Ten different disorder types were included in this review. These were selected as being the most dominant mental health disorders in the population. Due to the wide scope of our review, we acknowledge that we may have missed some studies for the disorder types of interest. Age-related disorders such as dementia were not included in this review as they were considered to reflect a different aspect of brain health.

From a clinical perspective, participants were typically recruited based on screening with DSM or ICD criteria for diagnosis, complemented by additional screening questionnaires. However, a handful of studies relied purely on screening questionnaires. The study participants also varied according to whether the clinical group was unmedicated (70%), defined as naïve or temporarily abstaining from taking medication for a variable length of time (12 h to 3 months) depending on the type of drug, medicated (5%) or included a mix of medicated and unmedicated patients (25%). Furthermore, although the majority of studies had specific inclusion and exclusion criteria, only a minority of studies specifically mention that they excluded patients with comorbidities, or specifically outlined the comorbidities in their patient group. The results from a particular disorder may therefore be influenced by other clinical comorbidities. Finally, the studies typically only report on spectral differences between groups and only 27% of studies provide insight into the relationship between the severity of the symptom score from the diagnosis questionnaires and the spectral bands.

#### Recording Configuration

A significant confound in the EEG space is the lack of standardization of hardware configurations and, in particular, the wide variety of different reference types used. Most common are linked ears (34%), average referencing (23%) and mastoids (15%). However, earlobes (14%), Cz (4%) and the nose (4%) are also used. The type of referencing used has a significant impact on the reported results, from the PSD and source localization (Trujillo et al., 2017) to functional connectivity (Huang et al., 2017) and various other aspects (e.g., Qin et al., 2010; Lei and Liao, 2017).

In addition, although the majority of studies covered the entire scalp, some studies chose to focus on midline sites. Only a proportion of studies reported results from individual electrodes (32%), with the majority choosing to focus on broad scalp regions (60%). In addition, some studies calculated the power spectrum using source localization techniques (e.g., LORETA) which may have resulted in a different regional profile from those studies focusing on the location of the electrodes on the scalp.

#### Processing of the Signal

The length of recordings was fairly consistent with a median of 5 min. However, often this entire recording window is not used but is divided up into artifact free segments that are epoched before the FFT computation is applied. The epoch length used for the FFT transform displays considerable variability (from 0.5 s to 600 s) with a median of 2.5 s. This variability is of concern as this can impact the window length utilized in the FFT algorithm and therefore the spreading or leakage across frequencies.

There are also inconsistencies in the methods used for identification of artifacts. This is sometimes done with methods such as Independent Component Analysis (ICA; Makeig et al., 1996; Vigário, 1997; Vigário et al., 2000; Jung et al., 2003) but many other techniques exist and many still use a manual or visually determined approach which can be highly inconsistent from “expert” to “expert” (see Urigüen and Garcia-Zapirain, 2015; Islam et al., 2016). These can result in substantial differences in the signal and therefore the spectral results.

The method used to determine the spectrum and different normalizations are another aspect of variability that can impact the magnitude of differences. There are presently a wide variety of software packages, algorithms and parameters used for computing the power spectrum. Software packages and functions include MATLAB/EEGLab Brainwave, Cadwell, sLORETA, eLORETA, RHYTHM, Neuroscan, Neuroguide, NXLink, Brain Vision Analyzer, Neurospeed, Persyst. While the FFT functions in these packages are roughly similar they do have differences in their default settings, and in some software the parameters used in the algorithm are not exposed and therefore not reported. Each function (for example spectrogram; pwelch algorithm; psd function; FFT function in MATLAB) further differ in their default settings with respect to the way the window length is selected, the overlap (here the studies range from 0 to 80%) and averaging (e.g., Bartlett or Welch method), and the windowing function used (e.g., Hamming or Hanning Window). All of these can make the difference between a small “significant” difference vs. a negative result (Keil et al., 2013). In addition, several studies did not provide any details about the parameters used, making it difficult to make a complete assessment of the consistency of methods in the field.

Finally, some studies report the differences in the absolute power and others report relative power which can also result in different outcomes, as we have seen above. However, as not all studies specifically mentioned whether they used absolute or relative power, for 29% of studies we had to infer which one was used.

#### Frequency Band Definition

**Figure 8:**
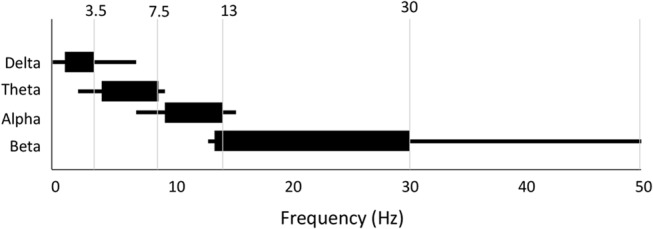
Variability in frequency band definition. Range of frequencies used for each band across all 184 studies. Thick line indicates most commonly used range.

Last, and perhaps most significant, there is a great deal of variability and confusion as to the specific frequency range that defines each band (Table 2). We show the more frequently used range as well as the entire range of definitions found in the reviewed literature in Figure 8. While alpha and theta were more consistent, delta could start anywhere from 0 Hz to 2 Hz and end anywhere from 3.5 Hz to 6 Hz. Meanwhile, beta could begin anywhere between 12 Hz and 15 Hz and end anywhere between 20 Hz and 50 Hz. Across all bands the most frequently used range was found in only 30- 50% of studies depending on the particular band. What one publication means by “delta,” or “beta” (etc.) is therefore not necessarily the same as what another publication means by the same terminology.

**Table 2:** Summary of frequency band parameters.

	% of Publications	Typical range (Hz)	Minimum start value (Hz)	Maximum end value (Hz)
Delta	70	1.3–3.5	0	6
Theta	84	4–7.5	2.5	8
Alpha	85	8–13	6	14
Beta	80	12.5–30	12	50
Gamma	18	30–40	20	100

Some of these differences arise on account of hardware configurations which apply different band pass filtering of the signal during preprocessing, forcibly defining the ranges and definition of the delta and gamma bands. Filters applied to resting state EEG data typically vary from 0.1 Hz to 1 Hz at the lower end to 40–100 Hz at the upper end. In addition, notch filters are often applied at 50 Hz and/or 60 Hz to filter out AC line noise.

Such differences in definition have enormous consequences for interpretation. Indeed, it’s conceivable that differences reported may disappear when moving the band windows slightly. Thus, the considerable variability and overlapping definitions across studies greatly diminishes the value of using the terminology of macro bands.

#### Reporting Omissions

One limitation of the literature was the possible bias towards positive results. For example, some studies did not include results for all bands specified in the methods. This could result in a number of unreported non-significant results that skew our analysis, particularly in the less frequently reported delta and beta bands.

Another inadequacy of this review relates to the sparse reporting of magnitudes of power change. Magnitude changes were calculated where the information was readily available, which was only in 40% of studies. The consequence of this is that the magnitude estimations are only based on a subset of studies, and do not necessarily reflect the complete picture. This is especially the case for ADHD (children) and OCD where the availability of magnitude data was considerably lower. In addition, this incomplete reporting of magnitudes across studies, and across individual electrodes also makes it challenging to study regional differences in spectral power in a consistent manner.

In summary, there are a number of dimensions of methodological variability and omissions that form limitations for this review and the field in general.

## Discussion

Our review describes reported differences in bands of the EEG power spectrum between controls and those with various psychiatric disorders including ADHD, schizophrenia, depression, bipolar disorder, anxiety, panic disorder, autism, PTSD, OCD and addiction. Across all disorders and conditions however, there was a wide range of often contradictory results for each frequency band (delta, theta, alpha, beta, gamma), although one result typically dominated. When considering the dominant results, the pattern that emerged is a tendency for higher levels in the low frequency bands (delta and theta) coupled with lower levels in the higher frequency bands (alpha, beta, gamma) across one group of disorders (ADHD, schizophrenia and OCD) relative to controls, and little to no difference in the power spectrum for others (addiction, PTSD and autism). Significant differences in this second set, when reported, were most often decreases in the higher frequency bands. Depression stood out as having a different pattern—an increase across the entire spectrum.

Across all disorders and conditions, the number of studies reporting the dominant result was on average 2.2 times the number of studies reporting other results and was similar across bands. In general, the eyes closed condition delivered more consistent results than the eyes open. Furthermore, while absolute power was most commonly reported, results were more consistent across studies for relative power. Across disorders and conditions, the validation score, a measure of how many participants and studies, on average, delivered the dominant result, was less than 250 for the majority of disorders. ADHD in children with eyes closed stood out as being by far the most studied and consistent in result, while schizophrenia, alcohol addiction, depression and PTSD with eyes closed followed by a substantial lag in being the next most reported and consistent in the literature. However, it is important to note that the majority of the ADHD (children) studies identified for this review were generated from a relatively small group of researchers (from the University of Wollongong) and the results from other research groups for ADHD are more variable. Other disorders and conditions were either too inconsistent or sparsely reported. The magnitude of significant results, when reported, was on average 34% across all bands and disorders for absolute power, and somewhat lower for relative power differences. Interestingly the magnitude of reported results was highest for schizophrenia (46%–53%) and lower than average for ADHD (11%–36%) and autism (11%–33%). Finally, the correlations between symptom severity and the power in any particular band was low for any brain regions reported and generally in the range of 0.3–0.5.

### Implications of These Results

The extreme lack of standardization across the field raises a strong caution to any clinical interpretation or application of current findings. From a purely methodological perspective, it is important that standards are imposed and adhered to in the research community. Particularly, we emphasize the need to use a standardized definition for each frequency band, based on the most commonly used non-overlapping frequencies: (delta: <4 Hz; theta: 4–7.5 Hz; alpha: 7.5–12.5 Hz; beta: 12.5–30 Hz; gamma: 30–40 Hz). Standardization of power spectrum computation, and the comparison of relative as well as absolute power are also essential. Absolute power, which relates to amplitude or magnitude of the signal, is more influenced by factors such as skull thickness and head geometry which vary considerably across people (Hagemann et al., 2008). These factors may be mitigated by the normalization used for relative power. Second, the eyes open paradigm is highly variable as visual input and attention can vary across subjects during the course of the experiment, pointing to eyes closed as a more uniform condition.

However, the generally common pattern across multiple disorders is an indication that individual frequency bands or even a pattern across frequency bands does not serve as a useful measure of distinction between disorders. It also strongly makes the case that studying individual disorders in isolation can be very misleading. For instance, a higher theta/beta ratio is considered an indicator of ADHD in children and even approved as a diagnostic marker by the FDA^8^. However, a similarly higher theta/beta ratio would be likely for schizophrenia and OCD as well. Psychiatric disorders are generally a loose set of symptoms that may overlap across disorders and there may be additional symptom comorbidities that are not accounted for in studies. Consequently, analysis based on specific symptoms and symptom clusters may yield more specific insights. This is particularly important to consider in the context of biomarkers based on the power spectrum.

It is also important to note that the patterns described across disorders are at a group level. For example, theta power was on average 27% higher in children diagnosed with ADHD vs. a control group. However, there was still substantial overlap in values between the groups. Further, the correlation values between symptom severity and power were typically around 0.4. This means that differences in frequency bands are not particularly useful for diagnosis at an individual level. With the wide variation in the power spectrum across normal populations and the lifespan (Haegens et al., 2014; Voytek et al., 2015; Hashemi et al., 2016; Arazi et al., 2017a,b; Parameshwaran and Thiagarajan, 2017a,b,c), it would be essential to look at larger sample sizes across multiple disorders, and with repeated recording sessions to control for both inter and intra person variability and parse out relationships to particular symptoms.

That said, in our view, these results along with the associated methodological concerns and limitations call for a new approach that goes beyond frequency bands to take into consideration new advances in our understanding of the power spectrum and new tools available for analysis.

### From Frequency Bands to Integrated Views: A Way Forward?

The Fourier transform which is used to describe the power spectrum was invented as a method of resolving sinusoids of different frequencies—an application of tremendous value in radio transmission. The EEG however is not a simple superposition of sinusoids of various frequencies. The power spectrum therefore should not be interpreted as such.

The dominant structure of the EEG power spectrum has been shown to be a decreasing function with lower power at higher frequencies that approximates a 1/f^γ^ pattern (Pritchard, 1992; Voytek et al., 2015). This is seen at various levels from surface measurements with microelectrodes (local field potentials or LFPs; Thiagarajan et al., 2010) and surface electrocorticographs or ECoG (Gao, 2016). The implication is that there is an inverse relationship between frequency and power across the measurable range and as the frequency increases, the power decreases. The steepness with which the power drops off as the frequency gets higher is represented by the exponent γ. This 1/f^γ^ structure has an important implication—that there is an underlying relationship or temporal correlations between the frequencies such that individual frequencies are not independent of one another (Milotti, 2002). We note however, that the origin and mechanisms of this structure is still very much a subject of debate as a frequency dependent filtering effect arising from the measurement cannot be ruled out (Bédard et al., 2006, 2017). Thus, if the spectrum best fits a 1/f function, the power of any individual frequency or range of frequency can be estimated if the exponent of the decay is known.

That said, there can be deviations from the 1/f spectrum. The most common such deviation are peaks that rises above the 1/f envelope, particularly with the eyes closed. The harmonic around 10 Hz (in the alpha range) is most commonly encountered, although it can sometimes occur at other frequencies and can be visualized in the autocorrelation of the signal. The presence of an alpha oscillation or harmonic peak is a feature that is distinct from the underlying 1/f envelope and should therefore be considered separately from the underlying envelope.

Specifically, we suggest reporting of metrics relating to the power spectrum in its entirety and identifying ways of identifying and separating periodicity from the 1/f background. There are a number of ways in which this can be done. The most obviously useful metric is estimation of the 1/f exponent γ (Voytek et al., 2015). This provides a consolidated view of the differences across bands and can be used to compute the difference between any two frequencies if desired. The 1/f decay exponent is best estimated in the range of 2–30 Hz where baseline drifts, line noise and distortions introduced by band pass filters have the least impact. Second, goodness of fit or other metrics that provide insight into deviations from the 1/f structure would also be informative. Recently new metrics have been proposed to provide a view into the degree of periodicity in different bands (Haller et al., 2018) and the harmonic component of the alpha band separately from the background envelope (Parameshwaran and Thiagarajan, 2017c). However, even while deeper views of the power spectrum could potentially provide better discrimination between disorders, this does not negate the impact of a lack of standardization in EEG measurement and methods used for signal preprocessing and computation of the power spectrum itself.

### Beyond the Power Spectrum

While there are ways to substantially improve our understanding of spectral properties in the context of psychiatric disorders, it is important to acknowledge that the power spectrum itself is a very general feature of the signal with few degrees of freedom. As we have seen it does not show significant difference across several disorders (autism, addiction, PTSD) and is not a reliable predictor of outcomes on an individual level (as shown by similarities of change across multiple disorders). Thus, on its own, it is not likely to provide fundamental discriminatory power between disorder types. Spatial views such as spectral coherence and hemispheric asymmetry can extend the scope of the spectral approach. However, spectral decomposition and spectral filtering by definition disregard relative phase information which may provide important discriminatory perspective.

The power spectrum might be thought of as analogous to describing a picture in terms of its color spectrum, or the relative distribution of red, blue and green pixels. While the color spectrum of an image can provide some general suggestion of the content of a picture (for example, higher blue on average means more sky, an increase in green means more nature), it loses all the relative spatial information that tells you what the picture actually is. The EEG power spectrum similarly loses relative temporal information, which at a more general level is one of the main advantages of EEG signal processing over other methods such as fMRI. Further, given that spectral decomposition is not instantaneous and utilized blocks of signal, and spectral filtering can distort the signal (Vanrullen, 2011; Acunzo et al., 2012; Rousselet, 2012; Widmann and Schröger, 2012), views such as spatial coherence are also limited in terms of their temporal resolution and insight into relative phase information. Using the same analogy of color, one might think of coherence, for example, as analogous to comparing the spatial positions of pixels in one narrow range of colors between successive image frames after some spatial blurring, while disregarding all the others.

We therefore suggest that more discriminating insights into differences between disorders are likely to be found in metrics that probe the temporal structure of the EEG signal as well as in novel connectivity measures. Numerous metrics have been proposed including various methods of assessing the entropy of the signal (Sabeti et al., 2009; Liang et al., 2015) and temporal memory (Jospin et al., 2007; Hardstone et al., 2012; Márton et al., 2014). However, this is a continually evolving field where new analytical tools are regularly being trialed. These should be increasingly embraced by EEG researchers involved in resting-state research who are looking to shift their approach away from spectral bands towards other potential methods which may offer greater clinical opportunities, applying these tools to their future and past datasets.

### A Call for Data Sharing and Sharable Analytical Pipelines

Finally, whether performing spectral analysis or exploring the signal using other methodologies, the primary issues are common. First, a lack of standardization of preprocessing steps and parameter choices within algorithms can result in a diversity of results that preclude easy comparison, and even appear contradictory. Second, small datasets limit the ability to determine meaningful results given the large diversity of human EEG dynamics. Consequently, we call for a concerted effort by the field to participate in open data efforts by sharing their raw data, and for those with analytical toolkits and analysis pipelines to make them available for the community. We emphasize the importance of sharing raw rather than preprocessed data given the numerous differences in preprocessing methodologies. We also emphasize the need for providing clear descriptions of the recording parameters such as the referencing, sampling rate and electrode characteristics.

While well-established databases specific for EEG are not yet available as they are for fMRI (e.g., openNeuro) some options presently exist and new ones are in development. For example, data can be shared in public repositories such as the National Institute of Mental Health Data Archive (NDA)^9^, Physionet^10^ and Zenodo^11^ or in the open EEG-specific database Brainbase which is presently in development (Thiagarajan, 2017). Similarly, open toolkits such as EEGLAB (MATLAB) and MNE (Python) are already available but complete pipelines are only now being established or are in development. In the meantime, tools can be shared on Github and other repositories and this should be made known through clear referencing in publications.

## Author Contributions

JN performed the literature search and discussed the results with TT. TT drafted the manuscript. TT and JN revised the manuscript, approved the final version, and agreed to be accountable for all aspects of the work.

## Conflict of Interest Statement

The authors declare that the research was conducted in the absence of any commercial or financial relationships that could be construed as a potential conflict of interest.

## Footnotes

^1^http://www.who.int/

^2^http://www.who.int/en/news-room/fact-sheets/detail/mental-disorders

^3^http://www.who.int/en/news-room/fact-sheets/detail/autism-spectrum-disorders

^4^https://www.accessdata.fda.gov/cdrh_docs/reviews/K112711.pdf

^5^https://www.ncbi.nlm.nih.gov/pubmed/

^6^http://www.who.int/features/qa/gaming-disorder/en/

^7^https://www.acns.org/practice/guidelines

^8^https://www.accessdata.fda.gov/cdrh_docs/reviews/K112711.pdf

^9^https://data-archive.nimh.nih.gov/

^10^https://www.physionet.org/

^11^https://zenodo.org/
